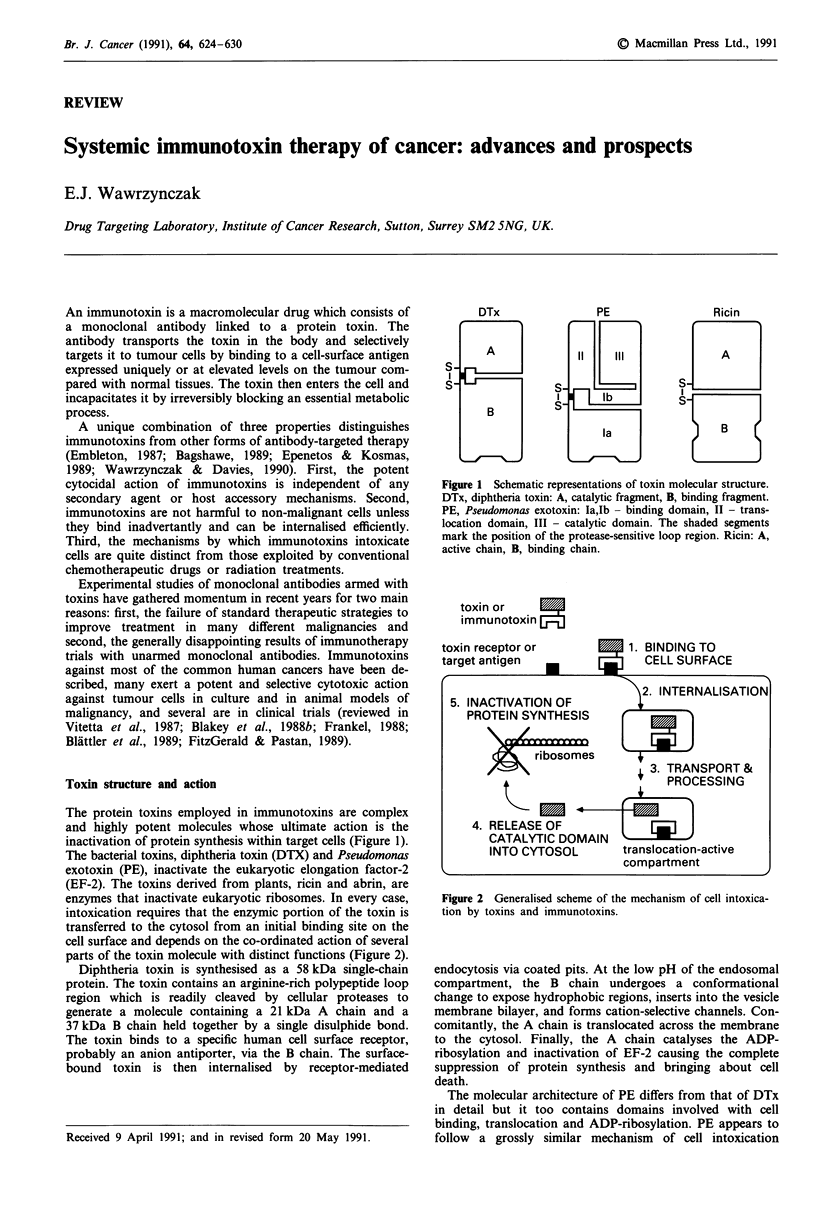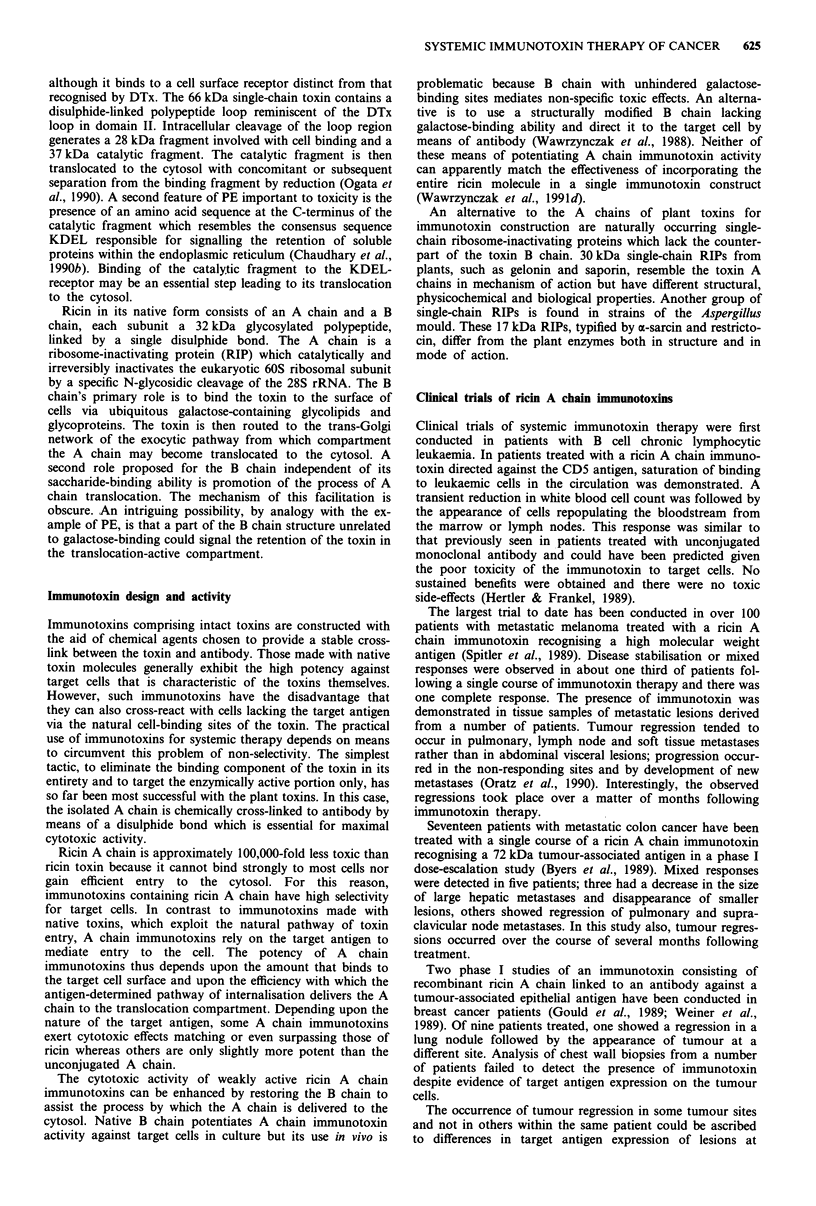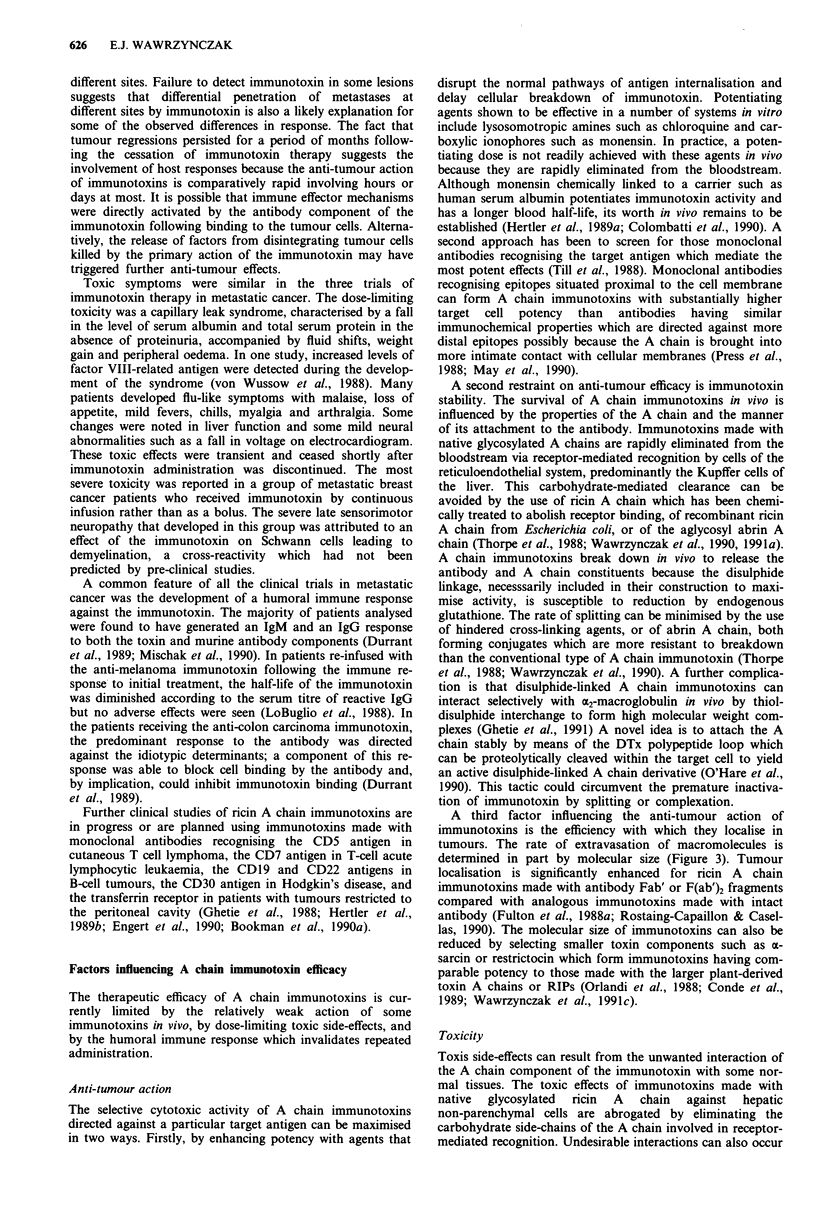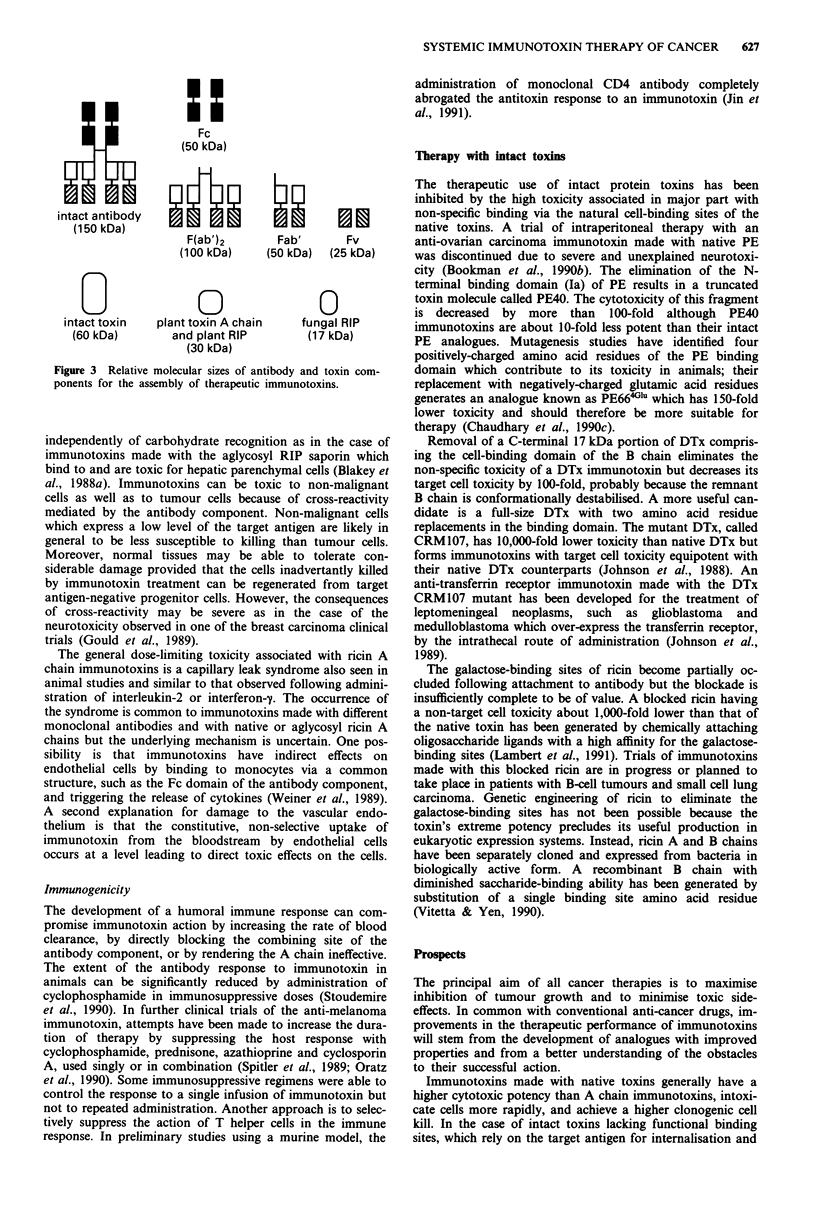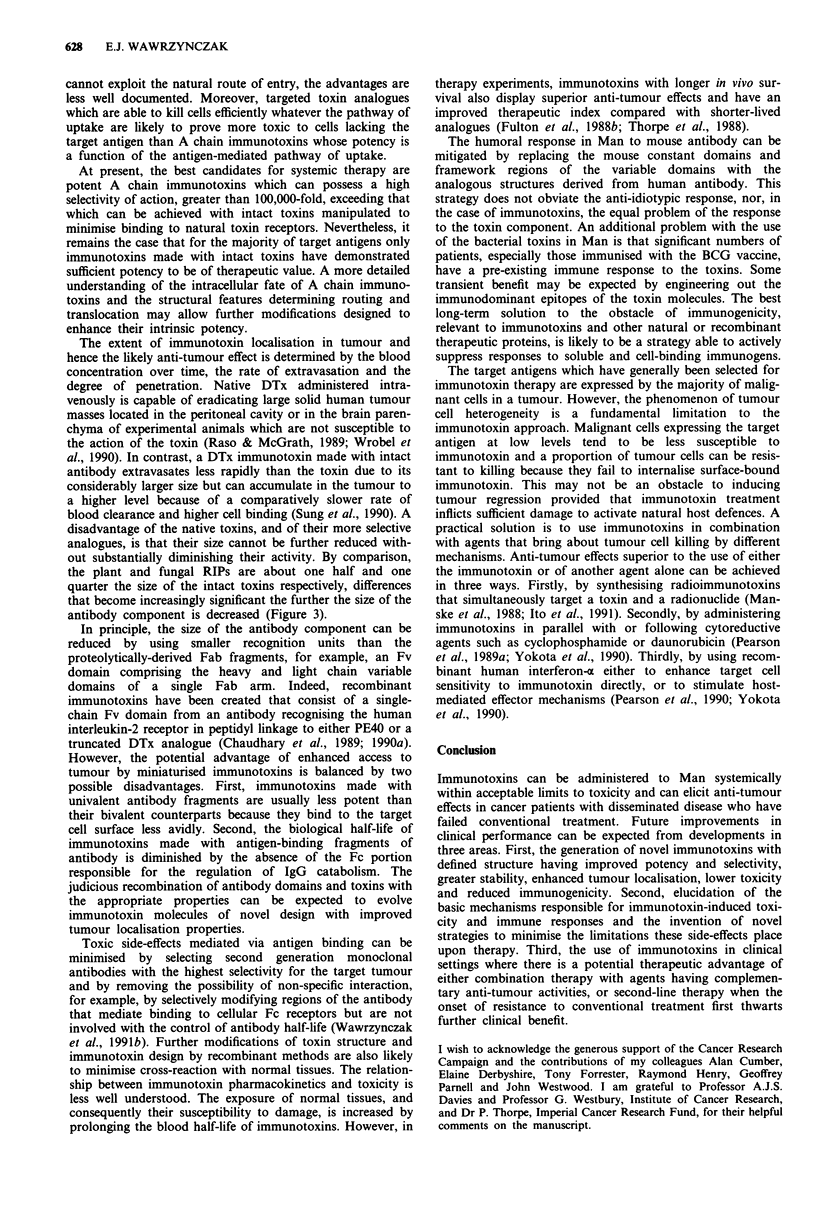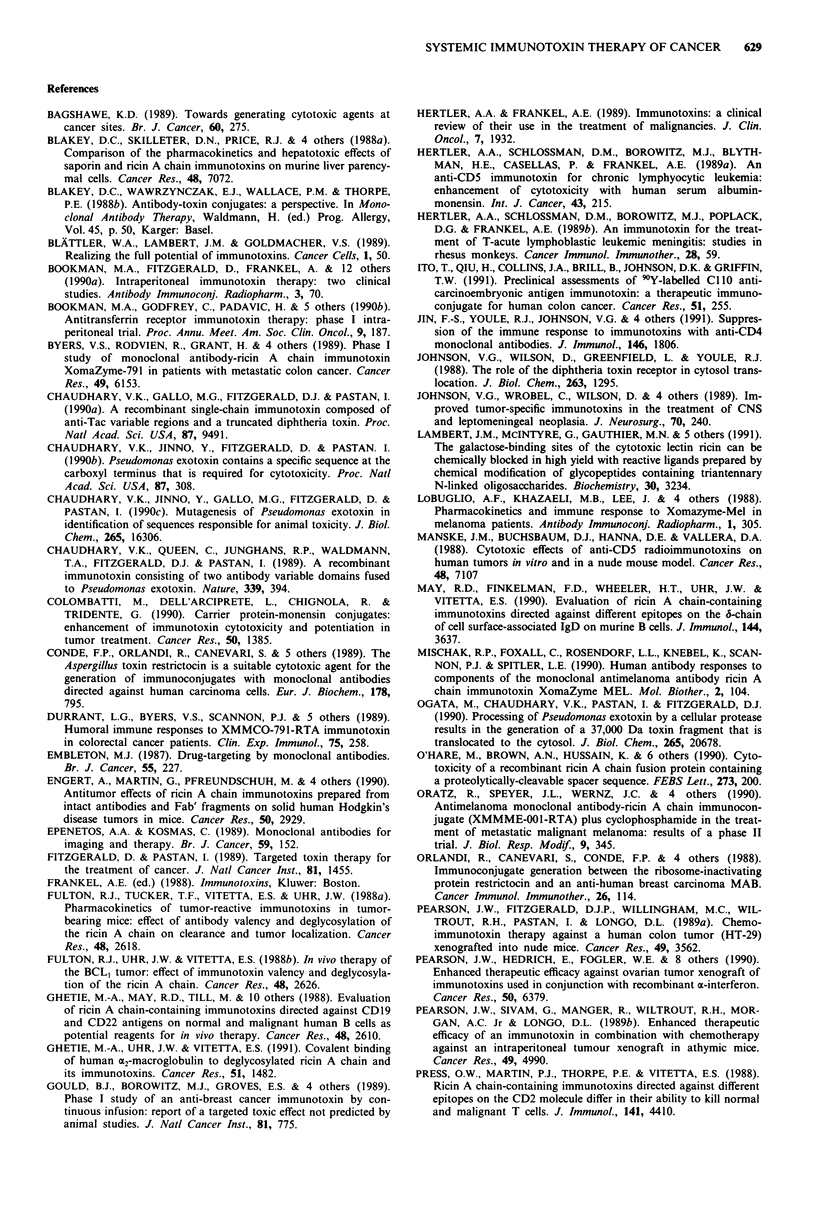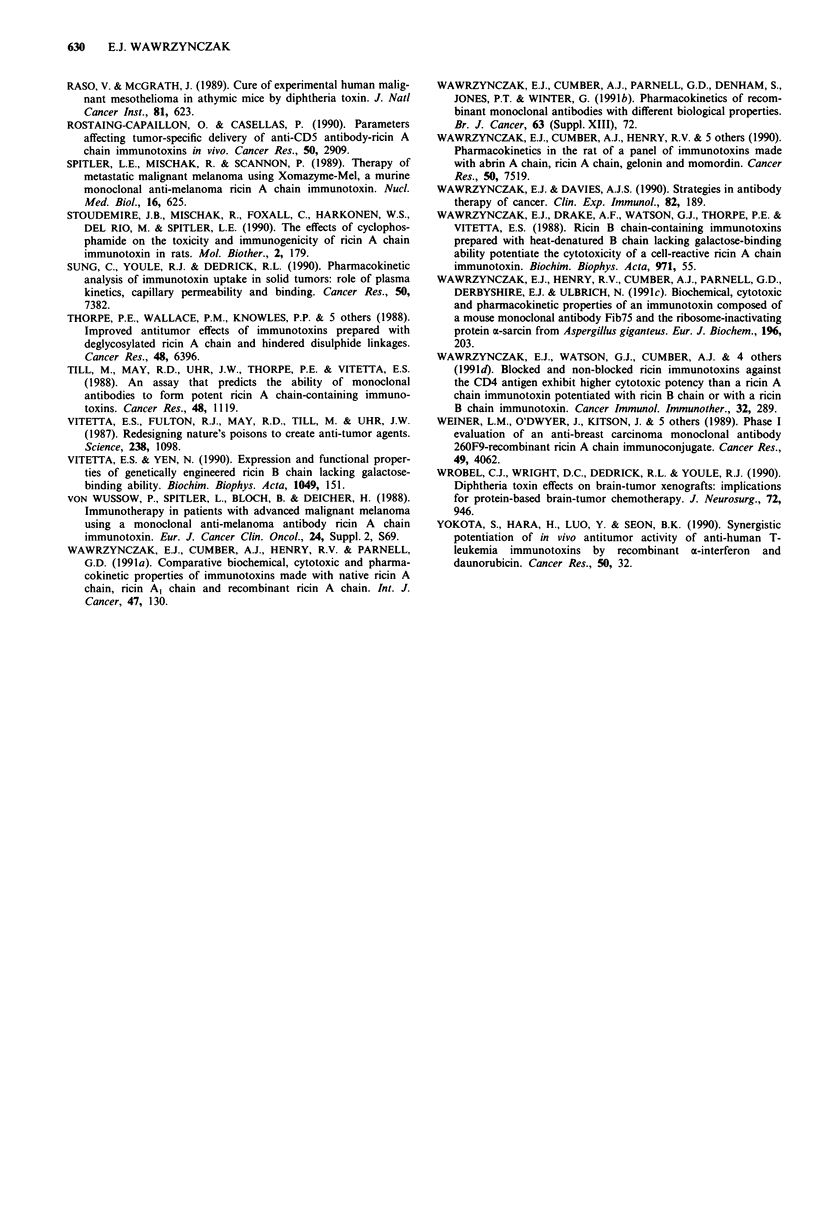# Systemic immunotoxin therapy of cancer: advances and prospects.

**DOI:** 10.1038/bjc.1991.374

**Published:** 1991-10

**Authors:** E. J. Wawrzynczak

**Affiliations:** Drug Targeting Laboratory, Institute of Cancer Research, Sutton, Surrey, UK.


*Br. J. Cancer* (1991), **64**, 624–630 © Macmillan Press Ltd., 1991

**REVIEW**

### Systemic immunotoxin therapy of cancer: advances and prospects

E.J. Wawrzynczak

*Drug Targeting Laboratory, Institute of Cancer Research, Sutton, Surrey SM2 5NG, UK.*

An immunotoxin is a macromolecular drug which consists of a monoclonal antibody linked to a protein toxin. The antibody transports the toxin in the body and selectively targets it to tumour cells by binding to a cell-surface antigen expressed uniquely or at elevated levels on the tumour compared with normal tissues. The toxin then enters the cell and incapacitates it by irreversibly blocking an essential metabolic process.

A unique combination of three properties distinguishes immunotoxins from other forms of antibody-targeted therapy (Embleton, 1987; Bagshawe, 1989; Epenetos & Kosmas, 1989; Wawrzynczak & Davies, 1990). First, the potent cytocidal action of immunotoxins is independent of any secondary agent or host accessory mechanisms. Second, immunotoxins are not harmful to non-malignant cells unless they bind inadvertantly and can be internalised efficiently. Third, the mechanisms by which immunotoxins intoxicate cells are quite distinct from those exploited by conventional chemotherapeutic drugs or radiation treatments.

Experimental studies of monoclonal antibodies armed with toxins have gathered momentum in recent years for two main reasons: first, the failure of standard therapeutic strategies to improve treatment in many different malignancies and second, the generally disappointing results of immunotherapy trials with unarmed monoclonal antibodies. Immunotoxins against most of the common human cancers have been described, many exert a potent and selective cytotoxic action against tumour cells in culture and in animal models of malignancy, and several are in clinical trials (reviewed in Vitetta *et al*., 1987; Blakey *et al*., 1988*b*; Frankel, 1988; Blättler *et al*., 1989; FitzGerald & Pastan, 1989).

### Toxin structure and action

The protein toxins employed in immunotoxins are complex and highly potent molecules whose ultimate action is the inactivation of protein synthesis within target cells (Figure 1). The bacterial toxins, diphtheria toxin (DTX) and *Pseudomonas* exotoxin (PE), inactivate the eukaryotic elongation factor-2 (EF-2). The toxins derived from plants, ricin and abrin, are enzymes that inactivate eukaryotic ribosomes. In every case, intoxication requires that the enzymic portion of the toxin is transferred to the cytosol from an initial binding site on the cell surface and depends on the co-ordinated action of several parts of the toxin molecule with distinct functions (Figure 2).

Diphtheria toxin is synthesised as a 58 kDa single-chain protein. The toxin contains an arginine-rich polypeptide loop region which is readily cleaved by cellular proteases to generate a molecule containing a 21 kDa A chain and a 37 kDa B chain held together by a single disulphide bond. The toxin binds to a specific human cell surface receptor, probably an anion antiporter, via the B chain. The surface-bound toxin is then internalised by receptor-mediated endocytosis via coated pits. At the low pH of the endosomal compartment, the B chain undergoes a conformational change to expose hydrophobic regions, inserts into the vesicle membrane bilayer, and forms cation-selective channels. Concomitantly, the A chain is translocated across the membrane to the cytosol. Finally, the A chain catalyses the ADP-ribosylation and inactivation of EF-2 causing the complete suppression of protein synthesis and bringing about cell death.

The molecular architecture of PE differs from that of DTx in detail but it too contains domains involved with cell binding, translocation and ADP-ribosylation. PE appears to follow a grossly similar mechanism of cell intoxication

**Figure 1** Schematic representations of toxin molecular structure. DTx, diphtheria toxin: **A**, catalytic fragment, **B**, binding fragment. PE, *Pseudomonas* exotoxin: Ia,Ib – binding domain, II – translocation domain, III – catalytic domain. The shaded segments mark the position of the protease-sensitive loop region. Ricin: **A**, active chain, **B**, binding chain.

**Figure 2** Generalised scheme of the mechanism of cell intoxication by toxins and immunotoxins.

Received 9 April 1991; and in revised form 20 May 1991.

although it binds to a cell surface receptor distinct from that recognised by DTx. The 66 kDa single-chain toxin contains a disulphide-linked polypeptide loop reminiscent of the DTx loop in domain II. Intracellular cleavage of the loop region generates a 28 kDa fragment involved with cell binding and a 37 kDa catalytic fragment. The catalytic fragment is then translocated to the cytosol with concomitant or subsequent separation from the binding fragment by reduction (Ogata *et al.*, 1990). A second feature of PE important to toxicity is the presence of an amino acid sequence at the C-terminus of the catalytic fragment which resembles the consensus sequence KDEL responsible for signalling the retention of soluble proteins within the endoplasmic reticulum (Chaudhary *et al.*, 1990*b*). Binding of the catalytic fragment to the KDEL-receptor may be an essential step leading to its translocation to the cytosol.

Ricin in its native form consists of an A chain and a B chain, each subunit a 32 kDa glycosylated polypeptide, linked by a single disulphide bond. The A chain is a ribosome-inactivating protein (RIP) which catalytically and irreversibly inactivates the eukaryotic 60S ribosomal subunit by a specific N-glycosidic cleavage of the 28S rRNA. The B chain's primary role is to bind the toxin to the surface of cells via ubiquitous galactose-containing glycolipids and glycoproteins. The toxin is then routed to the trans-Golgi network of the exocytic pathway from which compartment the A chain may become translocated to the cytosol. A second role proposed for the B chain independent of its saccharide-binding ability is promotion of the process of A chain translocation. The mechanism of this facilitation is obscure. An intriguing possibility, by analogy with the example of PE, is that a part of the B chain structure unrelated to galactose-binding could signal the retention of the toxin in the translocation-active compartment.

**Immunotoxin design and activity**

Immunotoxins comprising intact toxins are constructed with the aid of chemical agents chosen to provide a stable cross-link between the toxin and antibody. Those made with native toxin molecules generally exhibit the high potency against target cells that is characteristic of the toxins themselves. However, such immunotoxins have the disadvantage that they can also cross-react with cells lacking the target antigen via the natural cell-binding sites of the toxin. The practical use of immunotoxins for systemic therapy depends on means to circumvent this problem of non-selectivity. The simplest tactic, to eliminate the binding component of the toxin in its entirety and to target the enzymically active portion only, has so far been most successful with the plant toxins. In this case, the isolated A chain is chemically cross-linked to antibody by means of a disulphide bond which is essential for maximal cytotoxic activity.

Ricin A chain is approximately 100,000-fold less toxic than ricin toxin because it cannot bind strongly to most cells nor gain efficient entry to the cytosol. For this reason, immunotoxins containing ricin A chain have high selectivity for target cells. In contrast to immunotoxins made with native toxins, which exploit the natural pathway of toxin entry, A chain immunotoxins rely on the target antigen to mediate entry to the cell. The potency of A chain immunotoxins thus depends upon the amount that binds to the target cell surface and upon the efficiency with which the antigen-determined pathway of internalisation delivers the A chain to the translocation compartment. Depending upon the nature of the target antigen, some A chain immunotoxins exert cytotoxic effects matching or even surpassing those of ricin whereas others are only slightly more potent than the unconjugated A chain.

The cytotoxic activity of weakly active ricin A chain immunotoxins can be enhanced by restoring the B chain to assist the process by which the A chain is delivered to the cytosol. Native B chain potentiates A chain immunotoxin activity against target cells in culture but its use *in vivo* is problematic because B chain with unhindered galactose-binding sites mediates non-specific toxic effects. An alternative is to use a structurally modified B chain lacking galactose-binding ability and direct it to the target cell by means of antibody (Wawrzynczak *et al.*, 1988). Neither of these means of potentiating A chain immunotoxin activity can apparently match the effectiveness of incorporating the entire ricin molecule in a single immunotoxin construct (Wawrzynczak *et al.*, 1991*d*).

An alternative to the A chains of plant toxins for immunotoxin construction are naturally occurring single-chain ribosome-inactivating proteins which lack the counterpart of the toxin B chain. 30 kDa single-chain RIPs from plants, such as gelonin and saporin, resemble the toxin A chains in mechanism of action but have different structural, physicochemical and biological properties. Another group of single-chain RIPs is found in strains of the *Aspergillus* mould. These 17 kDa RIPs, typified by α-sarcin and restrictocin, differ from the plant enzymes both in structure and in mode of action.

**Clinical trials of ricin A chain immunotoxins**

Clinical trials of systemic immunotoxin therapy were first conducted in patients with B cell chronic lymphocytic leukaemia. In patients treated with a ricin A chain immunotoxin directed against the CD5 antigen, saturation of binding to leukaemic cells in the circulation was demonstrated. A transient reduction in white blood cell count was followed by the appearance of cells repopulating the bloodstream from the marrow or lymph nodes. This response was similar to that previously seen in patients treated with unconjugated monoclonal antibody and could have been predicted given the poor toxicity of the immunotoxin to target cells. No sustained benefits were obtained and there were no toxic side-effects (Hertler & Frankel, 1989).

The largest trial to date has been conducted in over 100 patients with metastatic melanoma treated with a ricin A chain immunotoxin recognising a high molecular weight antigen (Spitler *et al.*, 1989). Disease stabilisation or mixed responses were observed in about one third of patients following a single course of immunotoxin therapy and there was one complete response. The presence of immunotoxin was demonstrated in tissue samples of metastatic lesions derived from a number of patients. Tumour regression tended to occur in pulmonary, lymph node and soft tissue metastases rather than in abdominal visceral lesions; progression occurred in the non-responding sites and by development of new metastases (Oratz *et al.*, 1990). Interestingly, the observed regressions took place over a matter of months following immunotoxin therapy.

Seventeen patients with metastatic colon cancer have been treated with a single course of a ricin A chain immunotoxin recognising a 72 kDa tumour-associated antigen in a phase I dose-escalation study (Byers *et al.*, 1989). Mixed responses were detected in five patients; three had a decrease in the size of large hepatic metastases and disappearance of smaller lesions, others showed regression of pulmonary and supraclavicular node metastases. In this study also, tumour regressions occurred over the course of several months following treatment.

Two phase I studies of an immunotoxin consisting of recombinant ricin A chain linked to an antibody against a tumour-associated epithelial antigen have been conducted in breast cancer patients (Gould *et al.*, 1989; Weiner *et al.*, 1989). Of nine patients treated, one showed a regression in a lung nodule followed by the appearance of tumour at a different site. Analysis of chest wall biopsies from a number of patients failed to detect the presence of immunotoxin despite evidence of target antigen expression on the tumour cells.

The occurrence of tumour regression in some tumour sites and not in others within the same patient could be ascribed to differences in target antigen expression of lesions at different sites. Failure to detect immunotoxin in some lesions suggests that differential penetration of metastases at different sites by immunotoxin is also a likely explanation for some of the observed differences in response. The fact that tumour regressions persisted for a period of months following the cessation of immunotoxin therapy suggests the involvement of host responses because the anti-tumour action of immunotoxins is comparatively rapid involving hours or days at most. It is possible that immune effector mechanisms were directly activated by the antibody component of the immunotoxin following binding to the tumour cells. Alternatively, the release of factors from disintegrating tumour cells killed by the primary action of the immunotoxin may have triggered further anti-tumour effects.

Toxic symptoms were similar in the three trials of immunotoxin therapy in metastatic cancer. The dose-limiting toxicity was a capillary leak syndrome, characterised by a fall in the level of serum albumin and total serum protein in the absence of proteinuria, accompanied by fluid shifts, weight gain and peripheral oedema. In one study, increased levels of factor VIII-related antigen were detected during the development of the syndrome (von Wussow *et al.*, 1988). Many patients developed flu-like symptoms with malaise, loss of appetite, mild fevers, chills, myalgia and arthralgia. Some changes were noted in liver function and some mild neural abnormalities such as a fall in voltage on electrocardiogram. These toxic effects were transient and ceased shortly after immunotoxin administration was discontinued. The most severe toxicity was reported in a group of metastatic breast cancer patients who received immunotoxin by continuous infusion rather than as a bolus. The severe late sensorimotor neuropathy that developed in this group was attributed to an effect of the immunotoxin on Schwann cells leading to demyelination, a cross-reactivity which had not been predicted by pre-clinical studies.

A common feature of all the clinical trials in metastatic cancer was the development of a humoral immune response against the immunotoxin. The majority of patients analysed were found to have generated an IgM and an IgG response to both the toxin and murine antibody components (Durrant *et al.*, 1989; Mischak *et al.*, 1990). In patients re-infused with the anti-melanoma immunotoxin following the immune response to initial treatment, the half-life of the immunotoxin was diminished according to the serum titre of reactive IgG but no adverse effects were seen (LoBuglio *et al.*, 1988). In the patients receiving the anti-colon carcinoma immunotoxin, the predominant response to the antibody was directed against the idiotypic determinants; a component of this response was able to block cell binding by the antibody and, by implication, could inhibit immunotoxin binding (Durrant *et al.*, 1989).

Further clinical studies of ricin A chain immunotoxins are in progress or are planned using immunotoxins made with monoclonal antibodies recognising the CD5 antigen in cutaneous T cell lymphoma, the CD7 antigen in T-cell acute lymphocytic leukaemia, the CD19 and CD22 antigens in B-cell tumours, the CD30 antigen in Hodgkin's disease, and the transferrin receptor in patients with tumours restricted to the peritoneal cavity (Ghetie *et al.*, 1988; Hertler *et al.*, 1989*b*; Engert *et al.*, 1990; Bookman *et al.*, 1990*a*).

### Factors influencing A chain immunotoxin efficacy

The therapeutic efficacy of A chain immunotoxins is currently limited by the relatively weak action of some immunotoxins *in vivo*, by dose-limiting toxic side-effects, and by the humoral immune response which invalidates repeated administration.

### *Anti-tumour action*

The selective cytotoxic activity of A chain immunotoxins directed against a particular target antigen can be maximised in two ways. Firstly, by enhancing potency with agents that disrupt the normal pathways of antigen internalisation and delay cellular breakdown of immunotoxin. Potentiating agents shown to be effective in a number of systems *in vitro* include lysosomotropic amines such as chloroquine and carboxylic ionophores such as monensin. In practice, a potentiating dose is not readily achieved with these agents *in vivo* because they are rapidly eliminated from the bloodstream. Although monensin chemically linked to a carrier such as human serum albumin potentiates immunotoxin activity and has a longer blood half-life, its worth *in vivo* remains to be established (Hertler *et al.*, 1989*a*; Colombatti *et al.*, 1990). A second approach has been to screen for those monoclonal antibodies recognising the target antigen which mediate the most potent effects (Till *et al.*, 1988). Monoclonal antibodies recognising epitopes situated proximal to the cell membrane can form A chain immunotoxins with substantially higher target cell potency than antibodies having similar immunochemical properties which are directed against more distal epitopes possibly because the A chain is brought into more intimate contact with cellular membranes (Press *et al.*, 1988; May *et al.*, 1990).

A second restraint on anti-tumour efficacy is immunotoxin stability. The survival of A chain immunotoxins *in vivo* is influenced by the properties of the A chain and the manner of its attachment to the antibody. Immunotoxins made with native glycosylated A chains are rapidly eliminated from the bloodstream via receptor-mediated recognition by cells of the reticuloendothelial system, predominantly the Kupffer cells of the liver. This carbohydrate-mediated clearance can be avoided by the use of ricin A chain which has been chemically treated to abolish receptor binding, of recombinant ricin A chain from *Escherichia coli*, or of the aglycosyl abrin A chain (Thorpe *et al.*, 1988; Wawrzynczak *et al.*, 1990, 1991*a*). A chain immunotoxins break down *in vivo* to release the antibody and A chain constituents because the disulphide linkage, necesssarily included in their construction to maximise activity, is susceptible to reduction by endogenous glutathione. The rate of splitting can be minimised by the use of hindered cross-linking agents, or of abrin A chain, both forming conjugates which are more resistant to breakdown than the conventional type of A chain immunotoxin (Thorpe *et al.*, 1988; Wawrzynczak *et al.*, 1990). A further complication is that disulphide-linked A chain immunotoxins can interact selectively with $\alpha_2$-macroglobulin *in vivo* by thiol-disulphide interchange to form high molecular weight complexes (Ghetie *et al.*, 1991) A novel idea is to attach the A chain stably by means of the DTx polypeptide loop which can be proteolytically cleaved within the target cell to yield an active disulphide-linked A chain derivative (O'Hare *et al.*, 1990). This tactic could circumvent the premature inactivation of immunotoxin by splitting or complexation.

A third factor influencing the anti-tumour action of immunotoxins is the efficiency with which they localise in tumours. The rate of extravasation of macromolecules is determined in part by molecular size (Figure 3). Tumour localisation is significantly enhanced for ricin A chain immunotoxins made with antibody Fab' or $F(ab')_2$ fragments compared with analogous immunotoxins made with intact antibody (Fulton *et al.*, 1988*a*; Rostaing-Capaillon & Casellas, 1990). The molecular size of immunotoxins can also be reduced by selecting smaller toxin components such as α-sarcin or restrictocin which form immunotoxins having comparable potency to those made with the larger plant-derived toxin A chains or RIPs (Orlandi *et al.*, 1988; Conde *et al.*, 1989; Wawrzynczak *et al.*, 1991*c*).

### *Toxicity*

Toxis side-effects can result from the unwanted interaction of the A chain component of the immunotoxin with some normal tissues. The toxic effects of immunotoxins made with native glycosylated ricin A chain against hepatic non-parenchymal cells are abrogated by eliminating the carbohydrate side-chains of the A chain involved in receptor-mediated recognition. Undesirable interactions can also occur

**Figure 3** Relative molecular sizes of antibody and toxin components for the assembly of therapeutic immunotoxins.

independently of carbohydrate recognition as in the case of immunotoxins made with the aglycosyl RIP saporin which bind to and are toxic for hepatic parenchymal cells (Blakey *et al.*, 1988*a*). Immunotoxins can be toxic to non-malignant cells as well as to tumour cells because of cross-reactivity mediated by the antibody component. Non-malignant cells which express a low level of the target antigen are likely in general to be less susceptible to killing than tumour cells. Moreover, normal tissues may be able to tolerate considerable damage provided that the cells inadvertantly killed by immunotoxin treatment can be regenerated from target antigen-negative progenitor cells. However, the consequences of cross-reactivity may be severe as in the case of the neurotoxicity observed in one of the breast carcinoma clinical trials (Gould *et al.*, 1989).

The general dose-limiting toxicity associated with ricin A chain immunotoxins is a capillary leak syndrome also seen in animal studies and similar to that observed following administration of interleukin-2 or interferon-γ. The occurrence of the syndrome is common to immunotoxins made with different monoclonal antibodies and with native or aglycosyl ricin A chains but the underlying mechanism is uncertain. One possibility is that immunotoxins have indirect effects on endothelial cells by binding to monocytes via a common structure, such as the Fc domain of the antibody component, and triggering the release of cytokines (Weiner *et al.*, 1989). A second explanation for damage to the vascular endothelium is that the constitutive, non-selective uptake of immunotoxin from the bloodstream by endothelial cells occurs at a level leading to direct toxic effects on the cells.

### *Immunogenicity*

The development of a humoral immune response can compromise immunotoxin action by increasing the rate of blood clearance, by directly blocking the combining site of the antibody component, or by rendering the A chain ineffective. The extent of the antibody response to immunotoxin in animals can be significantly reduced by administration of cyclophosphamide in immunosuppressive doses (Stoudemire *et al.*, 1990). In further clinical trials of the anti-melanoma immunotoxin, attempts have been made to increase the duration of therapy by suppressing the host response with cyclophosphamide, prednisone, azathioprine and cyclosporin A, used singly or in combination (Spitler *et al.*, 1989; Oratz *et al.*, 1990). Some immunosuppressive regimens were able to control the response to a single infusion of immunotoxin but not to repeated administration. Another approach is to selectively suppress the action of T helper cells in the immune response. In preliminary studies using a murine model, the administration of monoclonal CD4 antibody completely abrogated the antitoxin response to an immunotoxin (Jin *et al.*, 1991).

### Therapy with intact toxins

The therapeutic use of intact protein toxins has been inhibited by the high toxicity associated in major part with non-specific binding via the natural cell-binding sites of the native toxins. A trial of intraperitoneal therapy with an anti-ovarian carcinoma immunotoxin made with native PE was discontinued due to severe and unexplained neurotoxicity (Bookman *et al.*, 1990*b*). The elimination of the N-terminal binding domain (Ia) of PE results in a truncated toxin molecule called PE40. The cytotoxicity of this fragment is decreased by more than 100-fold although PE40 immunotoxins are about 10-fold less potent than their intact PE analogues. Mutagenesis studies have identified four positively-charged amino acid residues of the PE binding domain which contribute to its toxicity in animals; their replacement with negatively-charged glutamic acid residues generates an analogue known as $PE66^{4Glu}$ which has 150-fold lower toxicity and should therefore be more suitable for therapy (Chaudhary *et al.*, 1990*c*).

Removal of a C-terminal 17 kDa portion of DTx comprising the cell-binding domain of the B chain eliminates the non-specific toxicity of a DTx immunotoxin but decreases its target cell toxicity by 100-fold, probably because the remnant B chain is conformationally destabilised. A more useful candidate is a full-size DTx with two amino acid residue replacements in the binding domain. The mutant DTx, called CRM107, has 10,000-fold lower toxicity than native DTx but forms immunotoxins with target cell toxicity equipotent with their native DTx counterparts (Johnson *et al.*, 1988). An anti-transferrin receptor immunotoxin made with the DTx CRM107 mutant has been developed for the treatment of leptomeningeal neoplasms, such as glioblastoma and medulloblastoma which over-express the transferrin receptor, by the intrathecal route of administration (Johnson *et al.*, 1989).

The galactose-binding sites of ricin become partially occluded following attachment to antibody but the blockade is insufficiently complete to be of value. A blocked ricin having a non-target cell toxicity about 1,000-fold lower than that of the native toxin has been generated by chemically attaching oligosaccharide ligands with a high affinity for the galactose-binding sites (Lambert *et al.*, 1991). Trials of immunotoxins made with this blocked ricin are in progress or planned to take place in patients with B-cell tumours and small cell lung carcinoma. Genetic engineering of ricin to eliminate the galactose-binding sites has not been possible because the toxin's extreme potency precludes its useful production in eukaryotic expression systems. Instead, ricin A and B chains have been separately cloned and expressed from bacteria in biologically active form. A recombinant B chain with diminished saccharide-binding ability has been generated by substitution of a single binding site amino acid residue (Vitetta & Yen, 1990).

### Prospects

The principal aim of all cancer therapies is to maximise inhibition of tumour growth and to minimise toxic side-effects. In common with conventional anti-cancer drugs, improvements in the therapeutic performance of immunotoxins will stem from the development of analogues with improved properties and from a better understanding of the obstacles to their successful action.

Immunotoxins made with native toxins generally have a higher cytotoxic potency than A chain immunotoxins, intoxicate cells more rapidly, and achieve a higher clonogenic cell kill. In the case of intact toxins lacking functional binding sites, which rely on the target antigen for internalisation and cannot exploit the natural route of entry, the advantages are less well documented. Moreover, targeted toxin analogues which are able to kill cells efficiently whatever the pathway of uptake are likely to prove more toxic to cells lacking the target antigen than A chain immunotoxins whose potency is a function of the antigen-mediated pathway of uptake.

At present, the best candidates for systemic therapy are potent A chain immunotoxins which can possess a high selectivity of action, greater than 100,000-fold, exceeding that which can be achieved with intact toxins manipulated to minimise binding to natural toxin receptors. Nevertheless, it remains the case that for the majority of target antigens only immunotoxins made with intact toxins have demonstrated sufficient potency to be of therapeutic value. A more detailed understanding of the intracellular fate of A chain immunotoxins and the structural features determining routing and translocation may allow further modifications designed to enhance their intrinsic potency.

The extent of immunotoxin localisation in tumour and hence the likely anti-tumour effect is determined by the blood concentration over time, the rate of extravasation and the degree of penetration. Native DTx administered intravenously is capable of eradicating large solid human tumour masses located in the peritoneal cavity or in the brain parenchyma of experimental animals which are not susceptible to the action of the toxin (Raso & McGrath, 1989; Wrobel *et al.*, 1990). In contrast, a DTx immunotoxin made with intact antibody extravasates less rapidly than the toxin due to its considerably larger size but can accumulate in the tumour to a higher level because of a comparatively slower rate of blood clearance and higher cell binding (Sung *et al.*, 1990). A disadvantage of the native toxins, and of their more selective analogues, is that their size cannot be further reduced without substantially diminishing their activity. By comparison, the plant and fungal RIPs are about one half and one quarter the size of the intact toxins respectively, differences that become increasingly significant the further the size of the antibody component is decreased (Figure 3).

In principle, the size of the antibody component can be reduced by using smaller recognition units than the proteolytically-derived Fab fragments, for example, an Fv domain comprising the heavy and light chain variable domains of a single Fab arm. Indeed, recombinant immunotoxins have been created that consist of a single-chain Fv domain from an antibody recognising the human interleukin-2 receptor in peptidyl linkage to either PE40 or a truncated DTx analogue (Chaudhary *et al.*, 1989; 1990*a*). However, the potential advantage of enhanced access to tumour by miniaturised immunotoxins is balanced by two possible disadvantages. First, immunotoxins made with univalent antibody fragments are usually less potent than their bivalent counterparts because they bind to the target cell surface less avidly. Second, the biological half-life of immunotoxins made with antigen-binding fragments of antibody is diminished by the absence of the Fc portion responsible for the regulation of IgG catabolism. The judicious recombination of antibody domains and toxins with the appropriate properties can be expected to evolve immunotoxin molecules of novel design with improved tumour localisation properties.

Toxic side-effects mediated via antigen binding can be minimised by selecting second generation monoclonal antibodies with the highest selectivity for the target tumour and by removing the possibility of non-specific interaction, for example, by selectively modifying regions of the antibody that mediate binding to cellular Fc receptors but are not involved with the control of antibody half-life (Wawrzynczak *et al.*, 1991*b*). Further modifications of toxin structure and immunotoxin design by recombinant methods are also likely to minimise cross-reaction with normal tissues. The relationship between immunotoxin pharmacokinetics and toxicity is less well understood. The exposure of normal tissues, and consequently their susceptibility to damage, is increased by prolonging the blood half-life of immunotoxins. However, in therapy experiments, immunotoxins with longer *in vivo* survival also display superior anti-tumour effects and have an improved therapeutic index compared with shorter-lived analogues (Fulton *et al.*, 1988*b*; Thorpe *et al.*, 1988).

The humoral response in Man to mouse antibody can be mitigated by replacing the mouse constant domains and framework regions of the variable domains with the analogous structures derived from human antibody. This strategy does not obviate the anti-idiotypic response, nor, in the case of immunotoxins, the equal problem of the response to the toxin component. An additional problem with the use of the bacterial toxins in Man is that significant numbers of patients, especially those immunised with the BCG vaccine, have a pre-existing immune response to the toxins. Some transient benefit may be expected by engineering out the immunodominant epitopes of the toxin molecules. The best long-term solution to the obstacle of immunogenicity, relevant to immunotoxins and other natural or recombinant therapeutic proteins, is likely to be a strategy able to actively suppress responses to soluble and cell-binding immunogens.

The target antigens which have generally been selected for immunotoxin therapy are expressed by the majority of malignant cells in a tumour. However, the phenomenon of tumour cell heterogeneity is a fundamental limitation to the immunotoxin approach. Malignant cells expressing the target antigen at low levels tend to be less susceptible to immunotoxin and a proportion of tumour cells can be resistant to killing because they fail to internalise surface-bound immunotoxin. This may not be an obstacle to inducing tumour regression provided that immunotoxin treatment inflicts sufficient damage to activate natural host defences. A practical solution is to use immunotoxins in combination with agents that bring about tumour cell killing by different mechanisms. Anti-tumour effects superior to the use of either the immunotoxin or of another agent alone can be achieved in three ways. Firstly, by synthesising radioimmunotoxins that simultaneously target a toxin and a radionuclide (Manske *et al.*, 1988; Ito *et al.*, 1991). Secondly, by administering immunotoxins in parallel with or following cytoreductive agents such as cyclophosphamide or daunorubicin (Pearson *et al.*, 1989*a*; Yokota *et al.*, 1990). Thirdly, by using recombinant human interferon-α either to enhance target cell sensitivity to immunotoxin directly, or to stimulate host-mediated effector mechanisms (Pearson *et al.*, 1990; Yokota *et al.*, 1990).

### Conclusion

Immunotoxins can be administered to Man systemically within acceptable limits to toxicity and can elicit anti-tumour effects in cancer patients with disseminated disease who have failed conventional treatment. Future improvements in clinical performance can be expected from developments in three areas. First, the generation of novel immunotoxins with defined structure having improved potency and selectivity, greater stability, enhanced tumour localisation, lower toxicity and reduced immunogenicity. Second, elucidation of the basic mechanisms responsible for immunotoxin-induced toxicity and immune responses and the invention of novel strategies to minimise the limitations these side-effects place upon therapy. Third, the use of immunotoxins in clinical settings where there is a potential therapeutic advantage of either combination therapy with agents having complementary anti-tumour activities, or second-line therapy when the onset of resistance to conventional treatment first thwarts further clinical benefit.

I wish to acknowledge the generous support of the Cancer Research Campaign and the contributions of my colleagues Alan Cumber, Elaine Derbyshire, Tony Forrester, Raymond Henry, Geoffrey Parnell and John Westwood. I am grateful to Professor A.J.S. Davies and Professor G. Westbury, Institute of Cancer Research, and Dr P. Thorpe, Imperial Cancer Research Fund, for their helpful comments on the manuscript.

### References

BAGSHAWE, K.D. (1989). Towards generating cytotoxic agents at cancer sites. *Br. J. Cancer*, **60**, 275.

BLAKEY, D.C., SKILLETER, D.N., PRICE, R.J. & 4 others (1988*a*). Comparison of the pharmacokinetics and hepatotoxic effects of saporin and ricin A chain immunotoxins on murine liver parencymal cells. *Cancer Res.*, **48**, 7072.

BLAKEY, D.C., WAWRZYNCZAK, E.J., WALLACE, P.M. & THORPE, P.E. (1988*b*). Antibody-toxin conjugates: a perspective. In *Monoclonal Antibody Therapy*, Waldmann, H. (ed.) Prog. Allergy, Vol. 45, p. 50, Karger: Basel.

BLÄTTLER, W.A., LAMBERT, J.M. & GOLDMACHER, V.S. (1989). Realizing the full potential of immunotoxins. *Cancer Cells*, **1**, 50.

BOOKMAN, M.A., FITZGERALD, D., FRANKEL, A. & 12 others (1990*a*). Intraperitoneal immunotoxin therapy: two clinical studies. *Antibody Immunoconj. Radiopharm.*, **3**, 70.

BOOKMAN, M.A., GODFREY, C., PADAVIC, H. & 5 others (1990*b*). Antitransferrin receptor immunotoxin therapy: phase I intraperitoneal trial. *Proc. Annu. Meet. Am. Soc. Clin. Oncol.*, **9**, 187.

BYERS, V.S., RODVIEN, R., GRANT, H. & 4 others (1989). Phase I study of monoclonal antibody-ricin A chain immunotoxin XomaZyme-791 in patients with metastatic colon cancer. *Cancer Res.*, **49**, 6153.

CHAUDHARY, V.K., GALLO, M.G., FITZGERALD, D.J. & PASTAN, I. (1990*a*). A recombinant single-chain immunotoxin composed of anti-Tac variable regions and a truncated diphtheria toxin. *Proc. Natl Acad. Sci. USA*, **87**, 9491.

CHAUDHARY, V.K., JINNO, Y., FITZGERALD, D. & PASTAN. I. (1990*b*). *Pseudomonas* exotoxin contains a specific sequence at the carboxyl terminus that is required for cytotoxicity. *Proc. Natl Acad. Sci. USA*, **87**, 308.

CHAUDHARY, V.K., JINNO, Y., GALLO, M.G., FITZGERALD, D. & PASTAN, I. (1990*c*). Mutagenesis of *Pseudomonas* exotoxin in identification of sequences responsible for animal toxicity. *J. Biol. Chem.*, **265**, 16306.

CHAUDHARY, V.K., QUEEN, C., JUNGHANS, R.P., WALDMANN, T.A., FITZGERALD, D.J. & PASTAN, I. (1989). A recombinant immunotoxin consisting of two antibody variable domains fused to *Pseudomonas* exotoxin. *Nature*, **339**, 394.

COLOMBATTI, M., DELL'ARCIPRETE, L., CHIGNOLA, R. & TRIDENTE, G. (1990). Carrier protein-monensin conjugates: enhancement of immunotoxin cytotoxicity and potentiation in tumor treatment. *Cancer Res.*, **50**, 1385.

CONDE, F.P., ORLANDI, R., CANEVARI, S. & 5 others (1989). The *Aspergillus* toxin restrictocin is a suitable cytotoxic agent for the generation of immunoconjugates with monoclonal antibodies directed against human carcinoma cells. *Eur. J. Biochem.*, **178**, 795.

DURRANT, L.G., BYERS, V.S., SCANNON, P.J. & 5 others (1989). Humoral immune responses to XMMCO-791-RTA immunotoxin in colorectal cancer patients. *Clin. Exp. Immunol.*, **75**, 258.

EMBLETON, M.J. (1987). Drug-targeting by monoclonal antibodies. *Br. J. Cancer*, **55**, 227.

ENGERT, A., MARTIN, G., PFREUNDSCHUH, M. & 4 others (1990). Antitumor effects of ricin A chain immunotoxins prepared from intact antibodies and Fab' fragments on solid human Hodgkin's disease tumors in mice. *Cancer Res.*, **50**, 2929.

EPENETOS, A.A. & KOSMAS, C. (1989). Monoclonal antibodies for imaging and therapy. *Br. J. Cancer*, **59**, 152.

FITZGERALD, D. & PASTAN, I. (1989). Targeted toxin therapy for the treatment of cancer. *J. Natl Cancer Inst.*, **81**, 1455.

FRANKEL, A.E. (ed.) (1988). *Immunotoxins*, Kluwer: Boston.

FULTON, R.J., TUCKER, T.F., VITETTA, E.S. & UHR, J.W. (1988*a*). Pharmacokinetics of tumor-reactive immunotoxins in tumor-bearing mice: effect of antibody valency and deglycosylation of the ricin A chain on clearance and tumor localization. *Cancer Res.*, **48**, 2618.

FULTON, R.J., UHR, J.W. & VITETTA, E.S. (1988*b*). *In vivo* therapy of the $BCL_1$ tumor: effect of immunotoxin valency and deglycosylation of the ricin A chain. *Cancer Res.*, **48**, 2626.

GHETIE, M.-A., MAY, R.D., TILL, M. & 10 others (1988). Evaluation of ricin A chain-containing immunotoxins directed against CD19 and CD22 antigens on normal and malignant human B cells as potential reagents for *in vivo* therapy. *Cancer Res.*, **48**, 2610.

GHETIE, M.-A., UHR, J.W. & VITETTA, E.S. (1991). Covalent binding of human $\alpha_2$-macroglobulin to deglycosylated ricin A chain and its immunotoxins. *Cancer Res.*, **51**, 1482.

GOULD, B.J., BOROWITZ, M.J., GROVES, E.S. & 4 others (1989). Phase I study of an anti-breast cancer immunotoxin by continuous infusion: report of a targeted toxic effect not predicted by animal studies. *J. Natl Cancer Inst.*, **81**, 775.

HERTLER, A.A. & FRANKEL, A.E. (1989). Immunotoxins: a clinical review of their use in the treatment of malignancies. *J. Clin. Oncol.*, **7**, 1932.

HERTLER, A.A., SCHLOSSMAN, D.M., BOROWITZ, M.J., BLYTHMAN, H.E., CASELLAS, P. & FRANKEL, A.E. (1989*a*). An anti-CD5 immunotoxin for chronic lymphyocytic leukemia: enhancement of cytotoxicity with human serum albumin-monensin. *Int. J. Cancer*, **43**, 215.

HERTLER, A.A., SCHLOSSMAN, D.M., BOROWITZ, M.J., POPLACK, D.G. & FRANKEL, A.E. (1989*b*). An immunotoxin for the treatment of T-acute lymphoblastic leukemic meningitis: studies in rhesus monkeys. *Cancer Immunol. Immunother.*, **28**, 59.

ITO, T., QIU, H., COLLINS, J.A., BRILL, B., JOHNSON, D.K. & GRIFFIN, T.W. (1991). Preclinical assessments of $^{90}$Y-labelled C110 anti-carcinoembryonic antigen immunotoxin: a therapeutic immunoconjugate for human colon cancer. *Cancer Res.*, **51**, 255.

JIN, F.-S., YOULE, R.J., JOHNSON, V.G. & 4 others (1991). Suppression of the immune response to immunotoxins with anti-CD4 monoclonal antibodies. *J. Immunol.*, **146**, 1806.

JOHNSON, V.G., WILSON, D., GREENFIELD, L. & YOULE, R.J. (1988). The role of the diphtheria toxin receptor in cytosol translocation. *J. Biol. Chem.*, **263**, 1295.

JOHNSON, V.G., WROBEL, C., WILSON, D. & 4 others (1989). Improved tumor-specific immunotoxins in the treatment of CNS and leptomeningeal neoplasia. *J. Neurosurg.*, **70**, 240.

LAMBERT, J.M., MCINTYRE, G., GAUTHIER, M.N. & 5 others (1991). The galactose-binding sites of the cytotoxic lectin ricin can be chemically blocked in high yield with reactive ligands prepared by chemical modification of glycopeptides containing triantennary N-linked oligosaccharides. *Biochemistry*, **30**, 3234.

LoBUGLIO, A.F., KHAZAELI, M.B., LEE, J. & 4 others (1988). Pharmacokinetics and immune response to Xomazyme-Mel in melanoma patients. *Antibody Immunoconj. Radiopharm.*, **1**, 305.

MANSKE, J.M., BUCHSBAUM, D.J., HANNA, D.E. & VALLERA, D.A. (1988). Cytotoxic effects of anti-CD5 radioimmunotoxins on human tumors *in vitro* and in a nude mouse model. *Cancer Res.*, **48**, 7107

MAY, R.D., FINKELMAN, F.D., WHEELER, H.T., UHR, J.W. & VITETTA, E.S. (1990). Evaluation of ricin A chain-containing immunotoxins directed against different epitopes on the $\delta$-chain of cell surface-associated IgD on murine B cells. *J. Immunol.*, **144**, 3637.

MISCHAK, R.P., FOXALL, C., ROSENDORF, L.L., KNEBEL, K., SCANNON, P.J. & SPITLER, L.E. (1990). Human antibody responses to components of the monoclonal antimelanoma antibody ricin A chain immunotoxin XomaZyme MEL. *Mol. Biother.*, **2**, 104.

OGATA, M., CHAUDHARY, V.K., PASTAN, I. & FITZGERALD, D.J. (1990). Processing of *Pseudomonas* exotoxin by a cellular protease results in the generation of a 37,000 Da toxin fragment that is translocated to the cytosol. *J. Biol. Chem.*, **265**, 20678.

O'HARE, M., BROWN, A.N., HUSSAIN, K. & 6 others (1990). Cytotoxicity of a recombinant ricin A chain fusion protein containing a proteolytically-cleavable spacer sequence. *FEBS Lett.*, **273**, 200.

ORATZ, R., SPEYER, J.L., WERNZ, J.C. & 4 others (1990). Antimelanoma monoclonal antibody-ricin A chain immunoconjugate (XMMME-001-RTA) plus cyclophosphamide in the treatment of metastatic malignant melanoma: results of a phase II trial. *J. Biol. Resp. Modif.*, **9**, 345.

ORLANDI, R., CANEVARI, S., CONDE, F.P. & 4 others (1988). Immunoconjugate generation between the ribosome-inactivating protein restrictocin and an anti-human breast carcinoma MAB. *Cancer Immunol. Immunother.*, **26**, 114.

PEARSON, J.W., FITZGERALD, D.J.P., WILLINGHAM, M.C., WILTROUT, R.H., PASTAN, I. & LONGO, D.L. (1989*a*). Chemo-immunotoxin therapy against a human colon tumor (HT-29) xenografted into nude mice. *Cancer Res.*, **49**, 3562.

PEARSON, J.W., HEDRICH, E., FOGLER, W.E. & 8 others (1990). Enhanced therapeutic efficacy against ovarian tumor xenograft of immunotoxins used in conjunction with recombinant $\alpha$-interferon. *Cancer Res.*, **50**, 6379.

PEARSON, J.W., SIVAM, G., MANGER, R., WILTROUT, R.H., MORGAN, A.C. Jr & LONGO, D.L. (1989*b*). Enhanced therapeutic efficacy of an immunotoxin in combination with chemotherapy against an intraperitoneal tumour xenograft in athymic mice. *Cancer Res.*, **49**, 4990.

PRESS, O.W., MARTIN, P.J., THORPE, P.E. & VITETTA, E.S. (1988). Ricin A chain-containing immunotoxins directed against different epitopes on the CD2 molecule differ in their ability to kill normal and malignant T cells. *J. Immunol.*, **141**, 4410.

RASO, V. & MCGRATH, J. (1989). Cure of experimental human malignant mesothelioma in athymic mice by diphtheria toxin. *J. Natl Cancer Inst.*, **81**, 623.

ROSTAING-CAPAILLON, O. & CASELLAS, P. (1990). Parameters affecting tumor-specific delivery of anti-CD5 antibody-ricin A chain immunotoxins *in vivo*. *Cancer Res.*, **50**, 2909.

SPITLER, L.E., MISCHAK, R. & SCANNON, P. (1989). Therapy of metastatic malignant melanoma using Xomazyme-Mel, a murine monoclonal anti-melanoma ricin A chain immunotoxin. *Nucl. Med. Biol.*, **16**, 625.

STOUDEMIRE, J.B., MISCHAK, R., FOXALL, C., HARKONEN, W.S., DEL RIO, M. & SPITLER, L.E. (1990). The effects of cyclophosphamide on the toxicity and immunogenicity of ricin A chain immunotoxin in rats. *Mol. Biother.*, **2**, 179.

SUNG, C., YOULE, R.J. & DEDRICK, R.L. (1990). Pharmacokinetic analysis of immunotoxin uptake in solid tumors: role of plasma kinetics, capillary permeability and binding. *Cancer Res.*, **50**, 7382.

THORPE, P.E., WALLACE, P.M., KNOWLES, P.P. & 5 others (1988). Improved antitumor effects of immunotoxins prepared with deglycosylated ricin A chain and hindered disulphide linkages. *Cancer Res.*, **48**, 6396.

TILL, M., MAY, R.D., UHR, J.W., THORPE, P.E. & VITETTA, E.S. (1988). An assay that predicts the ability of monoclonal antibodies to form potent ricin A chain-containing immunotoxins. *Cancer Res.*, **48**, 1119.

VITETTA, E.S., FULTON, R.J., MAY, R.D., TILL, M. & UHR, J.W. (1987). Redesigning nature's poisons to create anti-tumor agents. *Science*, **238**, 1098.

VITETTA, E.S. & YEN, N. (1990). Expression and functional properties of genetically engineered ricin B chain lacking galactose-binding ability. *Biochim. Biophys. Acta*, **1049**, 151.

VON WUSSOW, P., SPITLER, L., BLOCH, B. & DEICHER, H. (1988). Immunotherapy in patients with advanced malignant melanoma using a monoclonal anti-melanoma antibody ricin A chain immunotoxin. *Eur. J. Cancer Clin. Oncol.*, **24**, Suppl. 2, S69.

WAWRZYNCZAK, E.J., CUMBER, A.J., HENRY, R.V. & PARNELL, G.D. (1991*a*). Comparative biochemical, cytotoxic and pharmacokinetic properties of immunotoxins made with native ricin A chain, ricin $A_1$ chain and recombinant ricin A chain. *Int. J. Cancer*, **47**, 130.

WAWRZYNCZAK, E.J., CUMBER, A.J., PARNELL, G.D., DENHAM, S., JONES, P.T. & WINTER, G. (1991*b*). Pharmacokinetics of recombinant monoclonal antibodies with different biological properties. *Br. J. Cancer*, **63** (Suppl. XIII), 72.

WAWRZYNCZAK, E.J., CUMBER, A.J., HENRY, R.V. & 5 others (1990). Pharmacokinetics in the rat of a panel of immunotoxins made with abrin A chain, ricin A chain, gelonin and momordin. *Cancer Res.*, **50**, 7519.

WAWRZYNCZAK, E.J. & DAVIES, A.J.S. (1990). Strategies in antibody therapy of cancer. *Clin. Exp. Immunol.*, **82**, 189.

WAWRZYNCZAK, E.J., DRAKE, A.F., WATSON, G.J., THORPE, P.E. & VITETTA, E.S. (1988). Ricin B chain-containing immunotoxins prepared with heat-denatured B chain lacking galactose-binding ability potentiate the cytotoxicity of a cell-reactive ricin A chain immunotoxin. *Biochim. Biophys. Acta*, **971**, 55.

WAWRZYNCZAK, E.J., HENRY, R.V., CUMBER, A.J., PARNELL, G.D., DERBYSHIRE, E.J. & ULBRICH, N. (1991*c*). Biochemical, cytotoxic and pharmacokinetic properties of an immunotoxin composed of a mouse monoclonal antibody Fib75 and the ribosome-inactivating protein α-sarcin from *Aspergillus giganteus*. *Eur. J. Biochem.*, **196**, 203.

WAWRZYNCZAK, E.J., WATSON, G.J., CUMBER, A.J. & 4 others (1991*d*). Blocked and non-blocked ricin immunotoxins against the CD4 antigen exhibit higher cytotoxic potency than a ricin A chain immunotoxin potentiated with ricin B chain or with a ricin B chain immunotoxin. *Cancer Immunol. Immunother.*, **32**, 289.

WEINER, L.M., O'DWYER, J., KITSON, J. & 5 others (1989). Phase I evaluation of an anti-breast carcinoma monoclonal antibody 260F9-recombinant ricin A chain immunoconjugate. *Cancer Res.*, **49**, 4062.

WROBEL, C.J., WRIGHT, D.C., DEDRICK, R.L. & YOULE, R.J. (1990). Diphtheria toxin effects on brain-tumor xenografts: implications for protein-based brain-tumor chemotherapy. *J. Neurosurg.*, **72**, 946.

YOKOTA, S., HARA, H., LUO, Y. & SEON, B.K. (1990). Synergistic potentiation of *in vivo* antitumor activity of anti-human T-leukemia immunotoxins by recombinant α-interferon and daunorubicin. *Cancer Res.*, **50**, 32.